# Acutely blocking excessive mitochondrial fission prevents chronic neurodegeneration after traumatic brain injury

**DOI:** 10.1016/j.xcrm.2024.101715

**Published:** 2024-09-05

**Authors:** Preethy S. Sridharan, Yeojung Koh, Emiko Miller, Di Hu, Suwarna Chakraborty, Sunil Jamuna Tripathi, Teresa R. Kee, Kalyani Chaubey, Edwin Vázquez-Rosa, Sarah Barker, Hui Liu, Rose A. León-Alvarado, Kathryn Franke, Coral J. Cintrón-Pérez, Matasha Dhar, Min-Kyoo Shin, Margaret E. Flanagan, Rudolph J. Castellani, Tamar Gefen, Marina Bykova, Lijun Dou, Feixiong Cheng, Brigid M. Wilson, Hisashi Fujioka, David E. Kang, Jung-A.A. Woo, Bindu D. Paul, Xin Qi, Andrew A. Pieper

**Affiliations:** 1 Department of Psychiatry, Case Western Reserve University School of Medicine, Cleveland, OH, USA; 2 Brain Health Medicines Center, Harrington Discovery Institute, University Hospitals Cleveland Medical Center, Cleveland, OH, USA; 3 Geriatric Psychiatry, GRECC, Louis Stokes VA Medical Center, Cleveland, OH, USA; 4 Institute for Transformative Molecular Medicine, School of Medicine, Case Western Reserve University School of Medicine, Cleveland, OH, USA; 5 Department of Neurosciences, Case Western Reserve University School of Medicine, Cleveland, OH, USA; 6 Department of Pathology, Case Western Reserve University School of Medicine, Cleveland, OH, USA; 7 Department of Physiology and Biophysics, Case Western Reserve University School of Medicine, Cleveland, OH, USA; 8 Department of Pharmacology and Molecular Sciences, Johns Hopkins University School of Medicine, Baltimore, MD, USA; 9 Department of Molecular Medicine, USF Health College of Medicine, Tampa, FL, USA; 10 Earlham College Neuroscience Program, Richmond, IN, USA; 11 College of Pharmacy and Research Institute of Pharmaceutical Sciences, Seoul National University, Seoul 08226, Republic of Korea; 12 University of Texas Health Science Center at San Antonio, San Antonio, TX, USA; 13 Glenn Bigg’s Institute for Alzheimer’s & Neurodegenerative Diseases, University of Texas Health Science Center at San Antonio, San Antonio, TX, USA; 14 Department of Pathology, University of Texas Health Science Center at San Antonio, San Antonio, TX, USA; 15 Mesulam Center for Cognitive Neurology and Alzheimer’s Disease, Northwestern University Feinberg School of Medicine, Chicago, IL, USA; 16 Department of Pathology, Northwestern University Feinberg School of Medicine, Chicago, IL, USA; 17 Department of Psychiatry and Behavioral Sciences, Northwestern University Feinberg School of Medicine, Chicago, IL, USA; 18 Department of Regulatory Biology, Cleveland State University, Cleveland, OH, USA; 19 Genomic Medicine Institute, Lerner Research Institute, Cleveland Clinic, Cleveland, OH, USA; 20 Louis Stokes VA Medical Center, Cleveland, OH, USA; 21 Cryo-Electron Microscopy Core Facility, Case Western Reserve University School of Medicine, Cleveland, OH, USA; 22 Department of Psychiatry and Behavioral Sciences, Johns Hopkins University School of Medicine, Baltimore, MD, USA; 23 The Solomon H. Snyder Department of Neuroscience, Johns Hopkins University School of Medicine, Baltimore, MD, USA; 24 Lieber Institute for Brain Development, Baltimore, MD, USA

**Keywords:** traumatic brain injury, neurodegeneration, neuroprotection, mitochondria, Fis1, Drp1, mitochondrial fission, mitochondrial fusion, oxidative stress, blood-brain barrier

## Abstract

Progression of acute traumatic brain injury (TBI) into chronic neurodegeneration is a major health problem with no protective treatments. Here, we report that acutely elevated mitochondrial fission after TBI in mice triggers chronic neurodegeneration persisting 17 months later, equivalent to many human decades. We show that increased mitochondrial fission after mouse TBI is related to increased brain levels of mitochondrial fission 1 protein (Fis1) and that brain Fis1 is also elevated in human TBI. Pharmacologically preventing Fis1 from binding its mitochondrial partner, dynamin-related protein 1 (Drp1), for 2 weeks after TBI normalizes the balance of mitochondrial fission/fusion and prevents chronically impaired mitochondrial bioenergetics, oxidative damage, microglial activation and lipid droplet formation, blood-brain barrier deterioration, neurodegeneration, and cognitive impairment. Delaying treatment until 8 months after TBI offers no protection. Thus, time-sensitive inhibition of acutely elevated mitochondrial fission may represent a strategy to protect human TBI patients from chronic neurodegeneration.

## Highlights

• TBI in mice causes chronic neurodegeneration persisting 17 months later
• TBI in mice and humans acutely increases expression of mitochondrial fission 1 protein
• Acutely blocking mitochondrial fission after TBI prevents chronic neurodegeneration
• Equivalently blocking mitochondrial fission in chronic neurodegeneration has no benefit

## Graphical abstract

**Figure:**
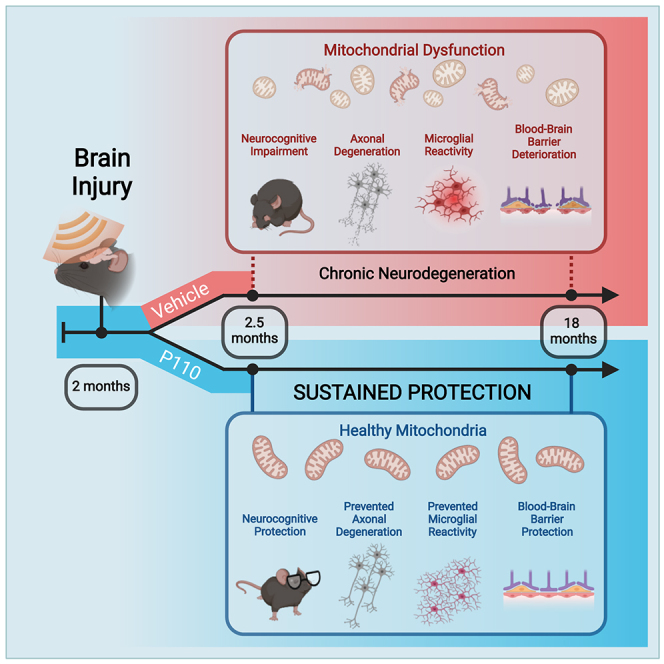

## Teaser

Sridharan et al. show that inhibiting aberrantly elevated mitochondrial fission for 2 weeks after traumatic brain injury (TBI) permanently normalizes mitochondrial fission/fusion and prevents chronically impaired bioenergetics, oxidative damage, blood-brain barrier deterioration, axonal degeneration, and cognitive impairment 17 months later, the equivalent of many decades in people.

## Introduction

Traumatic brain injury (TBI) is a leading cause of chronic neurodegeneration, and upward of 5 million Americans are currently living with consequent symptoms, such as chronically impaired cognition and increased risk of developing neurodegenerative diseases of aging, including Alzheimer’s disease (AD) and Parkinson’s disease (PD).^1^^,^^2^^,^^3^^,^^4^^,^^5^^,^^6^ Strikingly, the current annual incidence of TBI is ∼3.5 million in the USA and 70 million worldwide,^7^^,^^8^ and even subconcussive TBI can impair cognitive function.^9^ Unfortunately, there are currently no therapies that prevent chronic neurodegeneration after TBI. Our study was designed to address this unmet need.

TBI encompasses concussion, contusion, diffuse axonal injury, and open or closed head injury and is commonly sustained from falls, motor vehicle accidents, explosive forces, military combat, sports injuries, violent assaults, and other accidents. Thus, most forms of TBI are multifactorial. We considered this when designing a laboratory mouse model of multimodal TBI^10^ to test our hypothesis that mitochondrial fission would be elevated by TBI, akin to what is observed in other chronic neurodegenerative conditions.^11^^,^^12^^,^^13^^,^^14^ Specifically, we used a model of TBI that provides a comprehensive perspective on TBI’s impact by combining readily calibratable and reproducible aspects of global concussive injury, acceleration/deceleration trauma, and early blast wave exposure, the physics of which we have rigorously characterized.^10^ Key outcome parameters in this model align with the anomalies observed in human TBI patients, including early axonal degeneration followed by chronic neuronal cell death, cognitive and motor deficits, blood-brain barrier (BBB) deterioration, chronic neuroinflammation, systemic metabolic alterations in the blood, and blood biomarkers.^10^^,^^15^^,^^16^^,^^17^^,^^18^^,^^19^^,^^20^^,^^21^^,^^22^^,^^23^

Although the brain composes only 2% of the body’s mass, it comprises approximately 20% of the body’s total energy consumption.^24^ As neurons generate only 10% of total ATP through glycolysis,^25^ proper mitochondrial functioning is particularly critical for maintaining neuronal energy demand through the tricarboxylic acid cycle. Under healthy conditions, mitochondria maintain an equilibrium between fission and fusion that optimally serves the cell’s needs. Mitochondrial fission is mediated by translocation of the cytosolic GTPase dynamin-related protein 1 (Drp1) to the outer mitochondrial membrane, where it binds to adaptor proteins such as mitochondrial fission 1 (Fis1). While some mitochondrial fission is required for mitochondrial biogenesis and transport, aberrantly excessive mitochondrial fission is associated with excessive synthesis of reactive oxygen species (ROS), mitochondrial membrane depolarization, bioenergetic defects, mitophagy, and neuronal cell death.^11^ Importantly, pathologically elevated mitochondrial fission is a feature of many chronic neurodegenerative diseases, including AD, PD, and Huntington’s disease (HD).^11^^,^^12^^,^^13^^,^^14^ Thus, we hypothesized that mitochondrial fission might also be aberrantly elevated by TBI, and, if so, then its inhibition would mitigate the resulting chronic neurodegeneration.

Previous attempts have been made to block mitochondrial fission using the pharmacologic agent mitochondrial division inhibitor 1 (Mdivi-1), but this is a non-specific agent with numerous off-target effects, including inhibition of mitochondrial respiratory complexes and increased generation of ROS.^26^^,^^27^ Therefore, we chose to pharmacologically inhibit mitochondrial fission with P110, a selective small peptide inhibitor of pathologic mitochondrial fission that readily enters the brain as a transactivator of transcription (TAT) peptide^28^^,^^29^^,^^30^^,^^31^^,^^32^ and functions by blocking the binding interaction of Fis1 and Drp1 under stressed conditions.^32^ Protective efficacy of P110 requires the presence of Drp1 and does not affect expression of any fusion- or fission-related proteins.^28^^,^^32^ Treatment with P110 reduces mitochondrial fragmentation, mitochondrial damage, and tissue injury without affecting normal physiological mitochondrial dynamics and function.^13^^,^^28^^,^^33^^,^^34^^,^^35^ Notably, subcutaneous administration of P110 is protective in several preclinical animal models of neurodegenerative disease.^13^^,^^28^^,^^33^^,^^34^^,^^35^^,^^36^ In addition, acute or chronic administration of P110 has had no reported toxic effects in mice.^28^^,^^34^^,^^36^

Here, we show an early and rapid rise in Fis1 expression that corresponds with pathologically elevated mitochondrial fission after TBI. We also show that early transient treatment with P110 after TBI permanently restores normal homeostatic mitochondrial fission and blocks progression of acute TBI into chronic neurodegeneration in mice, even 17 months post-injury. This is the equivalent of decades in people.^37^ Measures of chronic neurodegeneration that are prevented by acute P110 treatment include impaired mitochondrial bioenergetics, oxidative damage, microglial activation and lipid droplet formation, BBB deterioration, neurodegeneration, and cognitive impairment. Delaying P110 treatment until after chronic neurodegeneration has developed, however, is not protective in any of these measures. Thus, early restoration of normal homeostatic mitochondrial fission after TBI prevents transition to chronic neurodegeneration.

## Results

### Elevated Fis1 protein and mitochondrial fission after TBI

**Figure 1:**
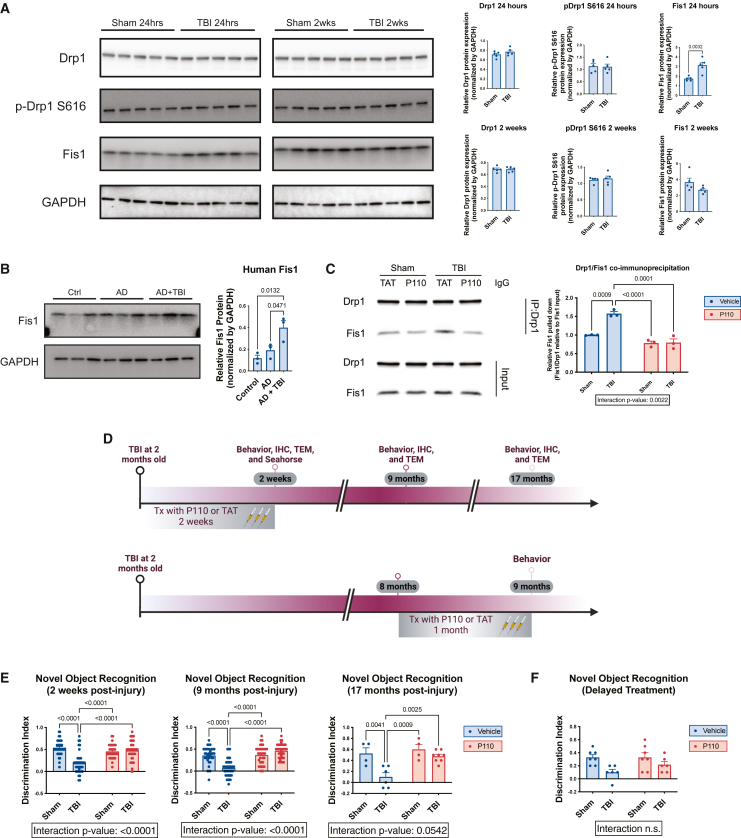
Prevention of chronic cognitive impairment after TBI by acute inhibition of pathologically excessive mitochondrial fission (A) Western blot and quantification of Drp1, phospho-Drp1 (pDrp1) S616, and Fis1 in mouse hippocampus at 24 h and 2 weeks post-injury show early elevation in Fis1 expression, but not Drp1 or pDrp1 S616 (*n* = 5 mice/group, two-tailed Student’s t test). (B) Western blot and quantification show that AD subjects with TBI history have elevated Fis1, but Fis1 is not elevated in subjects with AD alone (*n* = 3 patients/group, one-way ANOVA and Tukey’s post hoc analysis). (C) Co-immunoprecipitation of Drp1 with Fis1 shows increased interaction in the cerebellum of TBI TAT animals and reduced interaction in P110-treated animals (*n* = 3 mice/group). (D) Experimental schematic of injury and treatment strategy for early transient P110 treatment and experimental schematic of injury and treatment strategy for delayed transient P110 treatment. (E) Novel object recognition task at 2 weeks, 9 months, and 17 months post-injury shows significant cognitive impairment that is prevented by early transient P110 treatment (*n* = 35–40 mice/group for 2 weeks cohort, 36–40 mice/group for 9 months cohort, and *n* = 4–8 mice/group for 17 months cohort; two-way ANOVA and Tukey’s post hoc analysis). (F) Novel object recognition task at 9 months post-injury shows that cognitive impairment cannot be rescued with delayed transient P110 treatment (*n* = 6–7 mice/group; two-way ANOVA and Tukey’s post hoc analysis).

**Figure 2:**
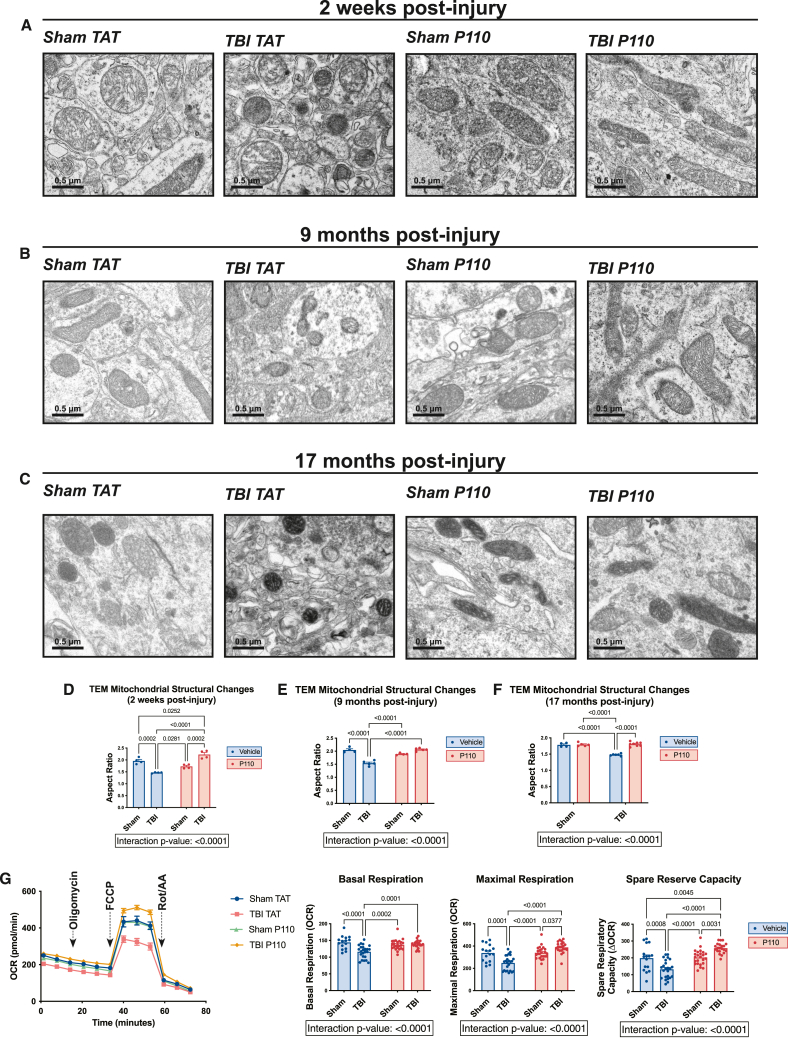
Prevention of chronic mitochondrial fragmentation and bioenergetic impairment after TBI by acute inhibition of pathologically excessive mitochondrial fission (A–C) Transmission electron microscopy (TEM) of hippocampal mitochondria collected 2 weeks, 9 months, and 17 months post-injury shows excessive mitochondrial fragmentation after TBI. Fragmentation is prevented with early transient P110 treatment. Structural changes are quantified by aspect ratio measurements. (D–F) Quantification of TEM images (*n* = 4–7 mice/group, with 700–1,000 mitochondria quantified and averaged per animal; two-way ANOVA and Tukey’s post hoc analysis). (G) Hippocampal synaptosome mitochondrial bioenergetics shows respiratory suppression 2 weeks after TBI, and P110 improves basal respiration, maximal respiration, and spare respiratory capacity (*n* = 16 samples (4 animals)/group; two-way ANOVA and Tukey’s post hoc analysis). Data are the means ± SEM. Two-way ANOVA tests for interaction between injury effect and treatment effect.

In contrast to the well-established aberrant elevation of mitochondrial fission due to increased Drp1 that has been reported in AD, PD, and HD,^13^^,^^35^^,^^38^^,^^39^ we did not observe a change in Drp1 or its activated form of phospho-Drp1 on serine 616 (S616) 24 h, 1 week, or 2 weeks after TBI (Figures 1A and S1A–S1C). However, we did observe significant early elevation of Fis1 24 h after TBI, modest elevation 1 week after TBI, and no elevation at 2 weeks (Figures 1A, S1B, and S1C). We also detected significantly elevated Fis1 expression in a small number of human subjects diagnosed with both AD and TBI, but not in those with AD alone (Figure 1B; Table S1). As detailed in the following, in our mouse model of TBI we additionally noted a significant increase in the number of smaller and rounded mitochondria after TBI, indicating excessive mitochondrial fission and fragmentation (Figures 2A–2F and S1D). These findings prompted us to hypothesize that inhibiting aberrantly high mitochondrial fission in this setting might be neuroprotective.

### Prevention of chronic cognitive impairment after TBI by acute inhibition of pathologically excessive mitochondrial fission

Co-immunoprecipitation showed increased binding of Drp1 and Fis1 after TBI in control TAT-treated animals, which was prevented in TBI mice receiving P110 treatment (Figures 1C and S2). Thus, to test the neuroprotective efficacy of blocking Fis1-Drp1 interaction after TBI, we subjected both male and female wild-type mice to either TBI or sham injury and subsequently treated them with either P110 or vehicle control TAT, starting 3 h after injury and continuing daily for 2 weeks (Figure 1D). Mice subjected to TBI and TAT treatment also demonstrated significant neurocognitive impairment in the novel object recognition task, which persisted chronically 6, 9, and 17 months later, whereas P110-treated mice were completely protected from TBI-induced cognitive impairment (Figures 1E and S3A). There were no observed effects of P110 treatment in sham-injured mice (Figures 1E and S3A). We next wondered whether delaying treatment until the chronic phase of TBI would also be effective. We chose to initiate a month-long regimen of P110 treatment at 8 months post-injury (Figure 1D), or 10 months of age, which corresponds roughly to a middle-aged human.^40^ With this treatment paradigm, there was no cognitive protection (Figure 1F). We did not observe any acute or chronic changes in anxiety-like (open field and light/dark tests) or depressive-like (forced swim test) behavior after TBI. We observed a very small effect of increased locomotor activity in males 6 months after TBI, which was normalized by early P110 treatment (Figures S3B–S3Q).

### Prevention of chronic mitochondrial fragmentation and impaired bioenergetics after TBI by acute inhibition of pathologically excessive mitochondrial fission

To determine how mitochondrial structure is altered as a function of TBI and selective inhibition of excessive mitochondrial fission, we collected brain tissue 2 weeks, 9 months, and 17 months after injury and imaged hippocampal ultrastructure using transmission electron microscopy (TEM). We conducted manual and blinded measurement of 700–1,000 mitochondria from every mouse of each group to determine aspect ratio, defined as the ratio between the maximal and minimal Feret’s diameter.^39^ An aspect ratio approaching 1 indicates greater sphericity, suggesting an increase in mitochondrial fragmentation, meaning an increase in fission-to-fusion ratio. This revealed mitochondrial fragmentation both acutely and chronically after TBI, which was prevented by treating mice with P110 for the 2 weeks immediately following TBI (Figures 2A–2F and S1D). Interestingly, at the 2-week time point, we observed a uniquely elongated phenotype in the TBI P110 group, with mitochondria longer than those in both the sham TAT and sham P110 groups (Figures 2A, 2D, and S1D). This illustrates that P110 exhibits specificity for damaged mitochondria without affecting healthy mitochondria.

We also interrogated the effect of TBI and P110 treatment on mitochondrial bioenergetics. Using hippocampal synaptosomes, we tested responsiveness to oligomycin, carbonyl cyanide 4-(trifluoromethoxy)phenylhydrazone (FCCP), and rotenone/antimycin to measure mitochondrial basal respiration, maximal respiration, and spare respiratory capacity. All measures were reduced by TBI and restored by P110 treatment (Figure 2G). Just as was observed with TEM, P110 treatment in the TBI condition caused mitochondrial function to improve beyond that of sham TAT and sham P110 groups. This further supports selective efficacy of P110 for conditions of pathologic mitochondrial fission.

### Prevention of chronic oxidative stress and microglial activation after TBI by acute inhibition of pathologically excessive mitochondrial fission

**Figure 3:**
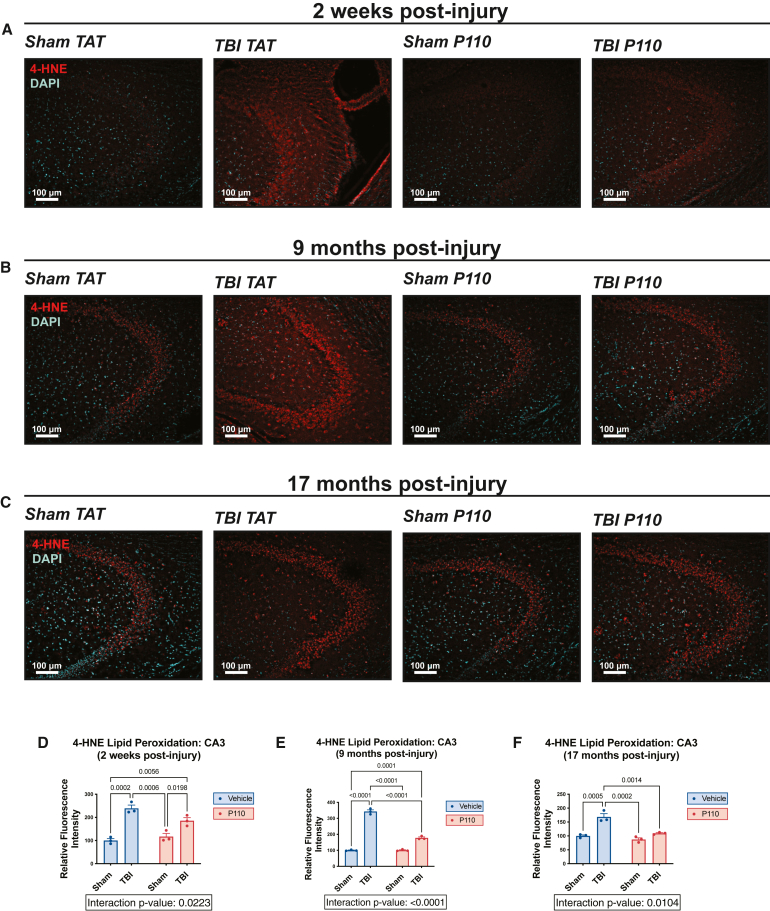
Prevention of chronic hippocampal lipid peroxidation after TBI by acute inhibition of excessive mitochondrial fission (A–C) Representative 4-HNE staining of the hippocampal CA3 region shows increased lipid peroxidation at 2 weeks, 9 months, and 17 months post-injury. Early transient P110 treatment prevents 4-HNE elevation. (D–F) Quantification of lipid peroxidation from CA3 region of hippocampus (*n* = 3 mice/group; two-way ANOVA and Tukey’s post hoc analysis).

Oxidative stress is intimately linked to mitochondrial dysfunction, inflammation, and neuronal damage and has been reported in several preclinical and clinical TBI studies.^41^^,^^42^^,^^43^^,^^44^^,^^45^^,^^46^^,^^47^^,^^48^^,^^49^ Specifically, the lipid-rich nature of the brain renders it highly vulnerable to lipid peroxidation, leading to synthesis of reactive aldehydes such as 4-hydroxy-2-nonenal (4-HNE),^50^^,^^51^ one of the most abundant and toxic products of lipid peroxidation.^52^^,^^53^ Importantly, 30% of the protein targets of 4-HNE are mitochondrial.^54^ 4-HNE disrupts mitochondrial bioenergetics by impairing the electron transport chain^55^^,^^56^^,^^57^ and perturbing the balance of mitochondrial fission/fusion^58^ and occurs in neurodegenerative disorders, including AD.^59^^,^^60^ Increased lipid peroxidation and oxidative stress have also been observed in preclinical TBI models and human TBI.^61^^,^^62^^,^^63^^,^^64^^,^^65^^,^^66^^,^^67^ To assess whether blocking excessive mitochondrial fission had any effect on oxidative stress and whether P110 could prevent TBI-induced lipid peroxidation, we immunohistochemically stained 4-HNE in brain slices derived from mice at 2 weeks, 9 months, and 17 months post-injury (Figures 3, S4, and S5). Elevated lipid peroxidation, as measured by 4-HNE staining, was observed in all sham TBI animals across the various ages, with maximal staining at 9 months. Lipid peroxidation after TBI was significantly decreased, however, by early P110 treatment at all time points. An interesting feature of the oxidative damage elicited by TBI was that lipid peroxidation was more pronounced in the CA3 region of the hippocampus than in the CA1 region or the dentate gyrus (Figures 3, S4, and S5). Although relative vulnerability of the CA3 region is well known,^68^ we report here the persistence of 4-HNE staining in the CA3 region at the very chronic time point of 17 months after TBI.

We next sought to extend these findings by using the thiobarbituric acid reactive substances (TBARSs) assay to quantify levels of malondialdehyde (MDA), an additional byproduct of lipid peroxidation that is typically elevated after brain injury in people.^63^^,^^64^^,^^65^^,^^66^ We observed significantly elevated MDA in the brains of mice at both 2 weeks and 9 months post-TBI, which was prevented by acute P110 treatment (Figure S6).

**Figure 4:**
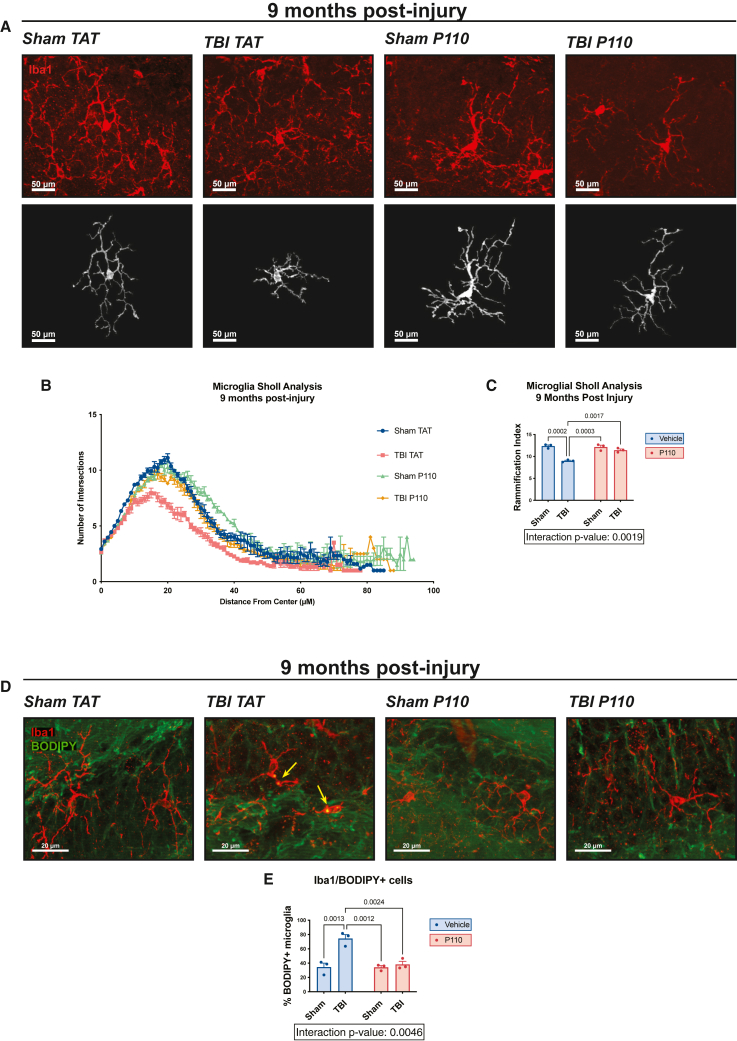
Prevention of chronic microglial hyperactivation and lipid droplet accumulation after TBI by acute inhibition of excessive mitochondrial fission (A) Representative Iba1 staining of hippocampus with skeletonized microglia. (B and C) Sholl analysis of hippocampal microglia 9 months post-injury shows a decrease in microglial ramification after TBI, which is prevented by early transient P110 treatment. (D) Representative BODIPY and Iba1 staining of hippocampus shows an increase in lipid droplet-positive microglia at 9 months post-injury, which is prevented by early transient P110 treatment. (E) Quantification of microglial lipid droplets in hippocampus (*n* = 3 mice/group; two-way ANOVA and Tukey’s post hoc analysis). Data are the means ± SEM. Two-way ANOVA tests for interaction between injury effect and treatment effect.

As an additional measure of oxidative-stress-related injury, we evaluated activation of microglia and the deposition of microglial lipid droplets, a toxic pro-inflammatory profile observed in aging and neurodegeneration.^69^ At 9 months post-injury, we observed significant reduction in microglial ramification in the hippocampus, suggesting an increase in microglial activation (Figures 4A–4C). Notably, mitochondria-derived ROS have been implicated in accumulation of microglial lipid droplets in both mice and *Drosophila melanogaster*.^70^ Interestingly, a loss of *Marf* (the *D. melanogaster* homolog of mitofusin-1 and mitofusin-2) is associated with ROS accumulation and subsequent lipid droplet formation, but Drp1 knockdown is not.^70^ These results suggest that a mitochondrial imbalance that favors fission promotes microglial lipid droplet formation, and neuron-specific mutations in these genes lead to lipid droplet formation only in microglia and not in neurons.^70^ Given the elevation of lipid peroxidation at all three measured time points after TBI in our model, and the clear protection of early transient P110 treatment in this parameter, we investigated the effect of TBI and P110 treatment on microglial lipid droplet accumulation by using the BODIPY label, as previously described.^69^ Concordantly, we observed accumulation of lipid droplets in hippocampal microglia 9 months post-injury, which was significantly reduced by early transient P110 treatment (Figures 3D and 3E).

### Prevention of chronic neurodegeneration and chronically impaired survival of young hippocampal neurons after TBI by acute inhibition of mitochondrial fission

**Figure 5:**
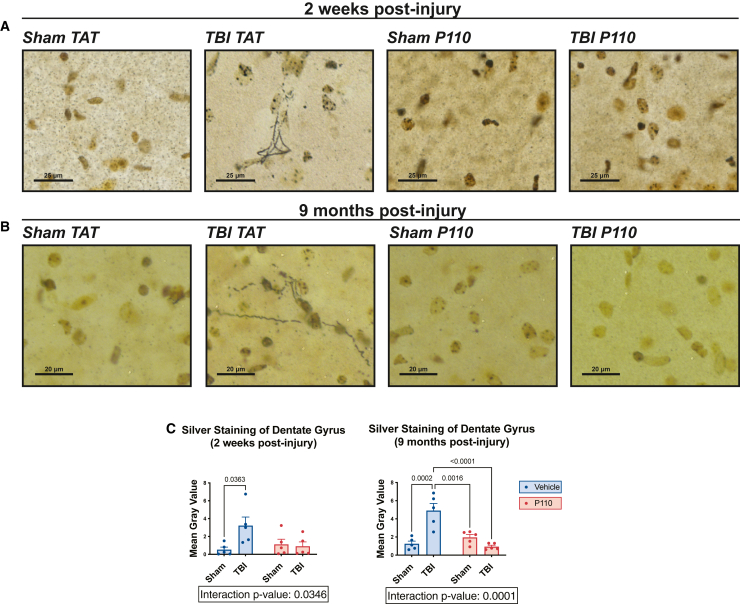
Prevention of chronic axonal neurodegeneration after TBI by acute inhibition of pathologically excessive mitochondrial fission (A and B) Representative images of neurodegeneration measured by silver staining of the hippocampus at 2 weeks and 9 months post-injury show significant protection with early transient P110 treatment. (C) Quantification of silver stain images (*n* = 5 mice/group, with 6 sections/animal counted and normalized to area; two-way ANOVA and Tukey’s post hoc analysis). Data are the means ± SEM. Two-way ANOVA tests for interaction between injury effect and treatment effect.

**Figure 6:**
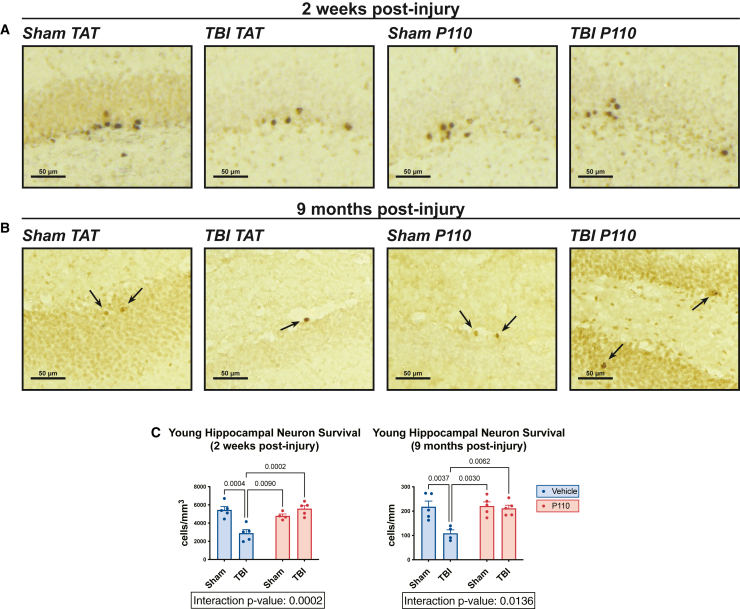
Prevention of chronically excessive death of young hippocampal neurons after TBI by acute inhibition of pathologically excessive mitochondrial fission (A and B) Representative images of BrdU-positive cell survival, which is impaired 2 weeks and 9 months post-injury and prevented by early transient P110 treatment. (C) BrdU-positive cell survival is impaired 9 months post-injury and prevented by early transient P110 treatment (*n* = 4–5 mice/group, with 6 sections/animal counted and normalized to area; two-way ANOVA and Tukey’s post hoc analysis). Data are the means ± SEM. Two-way ANOVA tests for interaction between injury effect and treatment effect.

To determine the extent of neurodegeneration as a function of TBI and pharmacologic treatment with P110, we used silver staining to label degenerating neurons.^17^^,^^71^^,^^72^ We observed elevated hippocampal silver staining at both 2 weeks and 9 months post-injury, which was significantly reduced by early treatment with P110 (Figures 5A–5C). We also investigated changes in the survival of young hippocampal neurons using 5-bromodeoxyuridine (BrdU) labeling, as TBI and other neurodegenerative conditions are characterized by reduced survival of these cells, which are generated by postnatal hippocampal neurogenesis. Mice were injected with a single bolus of BrdU either 2 or 3 weeks (for the acute and chronic experiments, respectively) prior to euthanasia to label dividing neural precursor cells in the hippocampal dentate gyrus, per established methods.^15^^,^^73^^,^^74^ Normally, most of these newborn cells die within the first few weeks, leaving only a fraction surviving to become young hippocampal neurons.^75^ We observed a significant reduction in survival of these cells both 2 weeks and 9 months after TBI, which was prevented by acute treatment with P110 (Figures 6A–6C).

### Prevention of chronic BBB deterioration after TBI by acute inhibition of excessive mitochondrial fission

**Figure 7:**
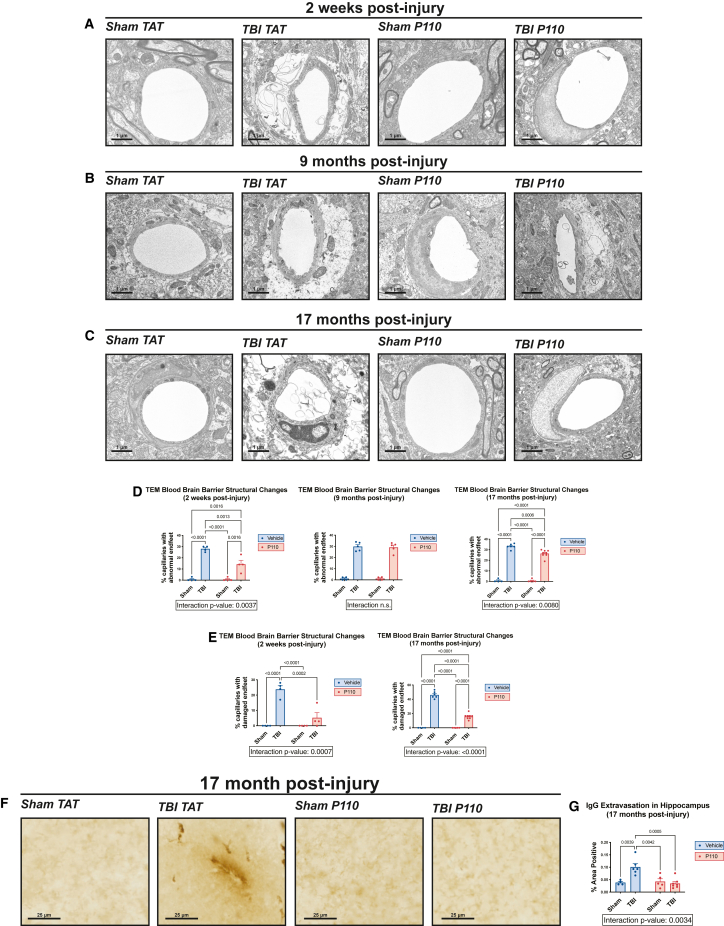
Prevention of chronic BBB deterioration after TBI is prevented by acute inhibition of excessive mitochondrial fission (A–C) Transmission electron microscopy shows significant deterioration of hippocampal astrocytic endfeet 2 weeks and 17 months post-injury, with minor swelling present at 9 months post-injury. Early transient P110 treatment mitigates deterioration of astrocytic endfeet. (D) Quantification of abnormal astrocytic endfeet, including both minor swelling and severe damage (*n* = 5–7 mice/group, with 30 capillaries counted per animal; two-way ANOVA and Tukey’s post hoc analysis). (E) Quantification of severely damaged astrocytic endfeet (*n* = 5–7 mice/group, with 30 capillaries counted per animal; two-way ANOVA and Tukey’s post hoc analysis). Data are the means ± SEM. Two-way ANOVA tests for interaction between injury effect and treatment effect. (F) Representative images of IgG staining show extravasation of IgG into brain parenchyma, indicating BBB deterioration. (G) Quantification of % hippocampal area positive for IgG extravasation (*n* = 3–7/group; two-way ANOVA and Tukey’s post ho*c* analysis).

We have previously reported that TBI causes early disruption of BBB permeability to 30 kDa dextran (6 h post-injury) and that this early leakiness is rectified at 24 h post-injury.^18^ The prominence of BBB deterioration in other preclinical models of TBI has also been recently reported by others.^76^ Here, we observed through TEM that, although endothelial barriers were intact 2 weeks after injury, there was significant astrocytic endfoot swelling along capillaries of the hippocampus. In addition, we observed frequent endfoot damage, defined as disruption of the astrocytic plasma membrane and destruction of organelles. Notably, animals acutely treated with P110 showed a reduction in both measurements of BBB damage (Figures 7A–7E). At 9 months post-injury, we did not observe significant endfoot damage, but both the TBI TAT and TBI P110 groups showed significant endfoot swelling (Figures 7B and 7D). By 17 months post-injury, astrocytic endfeet in the TBI TAT group had progressed to significant damage, which was prevented in the TBI P110 group (Figures 7C–7E). We then wondered whether this might be due to astrocytic reactivity after TBI, but we did not observe any changes in astrocyte reactivity at the chronic stage of injury (Figure S7). Next, we investigated whether the structural changes in the BBB corresponded to functional impairment. At the 17-month time point, we observed significant immunoglobulin G (IgG) extravasation into the parenchyma of the hippocampus, an indication of abnormal BBB permeability that was not observed in TBI animals receiving early P110 treatment (Figures 7F and 7G). Together, our results indicate a time-dependent progression of astrocyte endfoot dysfunction at the BBB after TBI, in which initial acute damage is restored to an extent but then resumes by 17 months after injury and is accompanied by functional impairment. Notably, early transient inhibition of mitochondrial fission mitigates this chronic progression of BBB damage after TBI.

## Discussion

Our results indicate that, as with other chronic neurodegenerative diseases, chronic neurodegeneration after TBI is driven by excessive mitochondrial fission and fragmentation. However, in contrast to AD, PD, and HD, in which this phenomenon is driven by elevated expression of Drp1, TBI is characterized by selective elevation of Fis1 in the absence of any changes to Drp1. As phospho-Drp1 is previously known to be elevated in human and mouse AD, this raises the possibility of synergistic effect resulting from simultaneous elevation of both phospho-Drp1 and Fis1 in the setting of combined TBI and AD and could be a contributing factor to how TBI accelerates the onset and severity of AD. Notably, the acutely significant increase in Fis1 24 h after TBI does not appear to persist after 2 weeks, and stabilization of excessive mitochondrial fission with P110 during this acute period is sufficient to produce long-lasting (more than 1 year) normalization of homeostatic mitochondrial fission. This is accompanied by protection from increased ROS and lipid peroxidation damage, BBB deterioration, axonal degeneration, microglial activation and lipid droplet accumulation, impaired survival of young hippocampal neurons, and neurocognitive impairment. Furthermore, at later time points after TBI when the process of chronic neurodegeneration has been established, P110 is not protective.

Recent evidence has shown that Fis1-mediated mitochondrial fission is specifically responsive to oxidative stress, altered mitochondrial membrane potential, and changes in intracellular calcium and pH.^77^ These properties of Fis1 may relate to the specificity that we observed in TBI and suggest that Fis1 represents a heretofore unidentified target for early therapeutic efforts after TBI. Given that aberrantly high mitochondrial fission in other forms of neurodegenerative disease, such as AD, PD, and HD, is associated with selectively elevated Drp1, future investigation will explore whether and how Fis1 or Drp1 are responsible for driving distinct mitochondrial deficits in different disease states. Understanding these disease-specific alterations could lead to more individualized treatment strategies for patients afflicted with various neurodegenerative pathologies.

One interesting observation in this study is that P110 is specifically effective at targeting injured mitochondria, in contrast to other mitochondrial fission inhibitors that have been tested in various neurodegenerative paradigms. For example, P110 treatment did not alter structure or bioenergetic function of hippocampal mitochondria in sham-injured mice but did elicit elongated mitochondria in TBI mice. P110 also improved mitochondrial bioenergetics of TBI mice beyond that of sham-injured mice. Thus, we conclude that P110 selectively targets damaged mitochondria to restore fission-fusion balance without disrupting healthy mitochondria or other vital cellular functions.

Our findings demonstrate that early, transient inhibition of mitochondrial fission after TBI prevents progression from acute to chronic neurodegeneration. This protective effect lasted until the longest time point examined, 17 months after TBI in mice, which is the equivalent of many decades in people. Later initiation of treatment with P110, after acute TBI had fully transitioned into chronic neurodegeneration, had no protective effect, indicating that mitochondrial fission-fusion pathology after TBI is important to address rapidly after injury. Interestingly, our group has recently demonstrated that a 1-month treatment with the brain NAD^+^/NADH-stabilizing agent P7C3-A20, initiated 1 year after TBI, completely halts neurodegeneration, restores structure and integrity of the BBB, attenuates neuroinflammation, and restores normal cognitive function.^18^^,^^78^ These findings, taken together with the results from the present study, suggest that promoting energetic homeostasis within the cell plays an important role in achieving effective therapy against neurodegeneration after TBI at all stages of disease and that different stages present different therapeutic opportunities.

Administration of P110 also significantly mitigated lipid peroxidation, which persisted well after a year post-TBI. We also reported amelioration of the extent of distribution and intensity of 4-HNE adducts over time following early transient blockade of excessive mitochondrial fission. This is further corroborated in our model by TBI-mediated elevation of MDA, a biomarker known to be elevated in people with TBI. These findings also correlate with our observations of TBI-mediated elevation in microglial activation and accumulation of lipid droplets 9 months post-injury, and likewise P110-mediated mitigation. Importantly, our results are concordant with prior reports that attenuating microglial activation in other models of TBI is protective in chronic injury.^79^

Lastly, in addition to the extensive long-term protection of neurons observed by silver staining of mature neurons and BrdU labeling of young newborn hippocampal neurons, we also report long-term protection against both acute and chronic changes in BBB structure and function. We note that the BBB deterioration that we observed by electron microscopy after TBI ranged from astrocytic endfoot swelling along capillaries to endfoot damage in the form of disrupted astrocytic plasma membrane and destruction of organelles. Not only are astrocytes an important component of the BBB but they are also widely abundant throughout the brain and crucial for virtually all aspects of brain function. While we did not specifically interrogate astrocytes in other regions of the brain, we note that others have shown that astrocytes play an important role in the pathophysiology of various forms of TBI.^76^^,^^80^^,^^81^^,^^82^^,^^83^ Given the protective efficacy of P110 that we observed for astrocytes at the BBB, and the previous report of the ability of P110 to protect astrocytes throughout the brain,^84^ it is reasonable to expect that P110 would also protect astrocytes in the various phases and forms of TBI that involve astrocytic pathology.

In conclusion, early stabilization of mitochondrial structure and function confers persistent restoration of normal brain function after TBI, as revealed by P110-mediated inhibition of TBI-induced mitochondrial fission. Addressing the high energy demand and disruption of metabolic homeostasis after brain injury in a timely manner, as shown here, could represent a converging neurotherapeutic target across many forms and stages of neurodegenerative disease.

### Limitations of the study

The phenomenon reported here has been reported in one model of TBI in mice (multimodal TBI), and the correlative finding of selectively elevated Fis1 in human TBI brain has been shown in a small number of cases. There are many different forms of TBI in people and many different models in mice, and it will be important in future work to establish whether our findings extend to the other forms and models of TBI. Given the multimodal nature of our injury model, we are optimistic that our results will prove relevant to a wide variety of other forms and models of TBI. However, injury-specific pathologic events and treatment approaches will undoubtedly be uncovered as the field advances. We also note that our human data demonstrating increased Fis1 expression in the brains of subjects with TBI and AD, but not AD alone, do not include isolated TBI brain samples, which we were unable to acquire. Additionally, treatment with P110 was limited to a single paradigm in which mice were treated daily for 2 weeks after injury. Additional work will be needed to further refine the time window during which aberrantly high mitochondrial fission provides an effective therapeutic target for preventing chronic neurodegeneration after TBI. For example, it would be valuable to know whether delaying treatment by a few days would still confer the same lasting therapeutic effect. Lastly, the mitochondrial data from the TEM and bioenergetic experiments were obtained exclusively from neurons. Although there was no gross reactivity observed in astrocytes, given the global nature of TBI and P110 treatment, further work will explore the role of mitochondrial fission in glial cells after injury. This will include interrogation of Fis1 and pDRP1 levels in discrete cell types in the brain as a function of time after TBI, as well as the effect of inducible cell-specific genetic elimination of Fis1 on both acute and chronic outcomes after TBI.

## Resource availability

### Lead contact

Further information and requests for resources and reagents should be directed to and will be fulfilled by the lead contact, Andrew A. Pieper (andrew.pieper@case.edu).

### Materials availability

This study did not generate new unique reagents.

### Data and code availability

All data reported in this paper will be shared by the lead contact upon request. This paper does not report the original code. Any additional information required to reanalyze the data reported in this paper is available from the lead contact upon request.

## Acknowledgments

A.A.P. was supported by the Valour Foundation. A.A.P. was also supported as the Rebecca E. Barchas, MD, DLFAPA, University Professor in Translational Psychiatry of Case Western Reserve University and the Morley-Mather Chair in Neuropsychiatry of University Hospitals of Cleveland Medical Center. A.A.P. and B.D.P. were supported by the 10.13039/100000968American Heart Association and Paul Allen Foundation Initiative in Brain Health and Cognitive Impairment (19PABH134580006) and by 10.13039/100000002NIH/10.13039/100000049NIA 1R01AG071512. A.A.P. also acknowledges support from the Department of Veterans Affairs Merit Award I01BX005976, 10.13039/100000002NIH/10.13039/100000057NIGMS RM1 GM142002, 10.13039/100000002NIH/10.13039/100000049NIA RO1AG066707, 10.13039/100000002NIH/10.13039/100000049NIA 1 U01 AG073323, the Louis Stokes VA Medical Center resources and facilities, the 10.13039/100000002Wick Foundation, and the Meisel & Pesses Family Foundation. X.Q. was supported by 10.13039/100000049NIH/NIA RO1AG065240, 10.13039/100000002NIH/10.13039/100000049NIA RF1AG074346, 10.13039/100000049NIH/NIA R01AG076051, and 10.13039/100000002NIH/10.13039/100000065NINDS R01NS115903. B.D.P. was supported by NIH NIDA grant P50 DA044123, 10.13039/100000002NIH 1R21AG073684-01, and the Catalyst Award from 10.13039/100007880Johns Hopkins University. D.E.K. and J.-A.A.W. were supported by 10.13039/100000002NIH/10.13039/100000049NIA 1R01AG067741-01 and 10.13039/100000002NIH/10.13039/100000065NINDS 1R01NS122218-A1. R.A.L.-A. was supported by the American Heart Association SURE Scholar Program. S.B. and E.M. were supported by the Alzheimer’s Disease Translational Data Science Training Program NIH T32 AGO71474. E.V.-R. was supported by Department of Defense Peer-Reviewed Alzheimer’s Research Program (PRARP) Award AZ210092 (W81XWH-22-1-0129). P.S.S. was supported by 10.13039/100000002NIH/10.13039/100000049NIA F30AG076183 and 10.13039/100000002NIH/10.13039/100000065NINDS T32NS077888. P.S.S. and S.B. were supported by Case Western Medical Scientist Training program NIH T32 GM007250. Y.K. was supported by NIH/NIA F99 AGO83111. M.-K.S. was supported by the New Faculty Startup Fund (370C-20220110), Creative-Pioneering Researchers Program (370C-20230108), and a research grant (370C-20240120) from Seoul National University. M.-K.S. also acknowledges support from the National Research Foundation of Korea (RS-2023-00209597, RS-2024-00352229) and donors of Alzheimer’s Disease Research, a program of BrightFocus Foundation (A2019551F). M.E.F., R.J.C., and T.G. were supported by grants from the 10.13039/100000049NIA (P30AG072977, R01AG062566). We also thank the Translational Research Shared Resource of the Case Comprehensive Cancer Center (10.13039/100000002NIH/10.13039/100000054NCI P30 CA43703) for the use of the Seahorse analyzer. We also acknowledge BioRender.com, a service we used to design our schematic figures.

## Author contributions

P.S.S., B.D.P., D.E.K., J.-A.A.W., X.Q., and A.A.P. designed experiments. P.S.S., B.D.P., X.Q., and A.A.P. wrote the manuscript, with input from all authors. All authors critically reviewed the manuscript. P.S.S., Y.K., E.M., S.C., S.J.T., T.R.K., K.C., E.V.-R., S.B., R.A.L.-A., K.F., C.J.C.-P., M.D., M.-K.S., M.B., L.D., F.C., H.F., D.E.K., J.-A.A.W., B.D.P., X.Q., and A.A.P. conducted experimental work. S.C., S.J.T., and B.D.P. collaborated on oxidative damage studies. T.R.K., D.E.K., and J.-A.A.W. collaborated on mitochondrial bioenergetics studies. M.E.F., R.J.C., and T.G. supplied human brain tissue. B.M.W. directed statistical analysis of the data. H.F. collaborated on electron microscopy studies. X.Q. and A.A.P. are the guarantors of this work and, as such, had full access to all the data in the study and take responsibility for the integrity of the data and the accuracy of the data analysis.

## Declaration of interests

X.Q. is an inventor of P110 and holds patents related to P110.

## STAR★Methods

### Key resources table

**Table:** 

REAGENT or RESOURCE	SOURCE	IDENTIFIER
**Antibodies**		

Mouse monoclonal anti-Drp1	BD Transduction	Cat# 611113
Rabbit polyclonal anti-Drp1S616	Cell Signaling Technology	Cat# 3455S
Rabbit polyclonal anti-Fis1	Proteintech	Cat# 10956-1-AP; RRID:AB_2102532
Rabbit monoclonal anti-GAPDH	Cell Signaling Technology	Cat#2118S
Mouse monoclonal anti-GFAP (ASTRO6)	Thermo Fisher Scientific	Cat# MA5-12023; RRID:AB_10984338
Rabbit polyclonal anti-Iba1	FUJIFILM Wako Shibayagi	Cat# 019-19741
Mouse monoclonal anti-NeuN	Millipore	Cat# MAB377; RRID:AB_2298772
Mouse monoclonal anti- 5-bromodeoxyuridine	Sigma-Aldrich	Cat# SAB4700630; RRID:AB_10896613
Rabbit polyclonal anti-4 hydroxynonenal (HNE) antiserum	Alpha Diagnostic International	Cat# HNE11-S, RRID:AB_2629282
Rabbit polyclonal anti-Nitrotyrosine Antibody	Millipore Sigma	Cat# AB5411, RRID:AB_177459
Goat polyclonal anti-rabbit IgG, HRP-linked	Cell Signaling Technology	Cat# 7074; RRID:AB_2099233
Horse polyclonal anti-mouse IgG, HRP-linked	Cell Signaling Technology	Cat# 7076; RRID:AB_330924
Alexa Fluor 488 Goat anti-Mouse IgG (H+L)	Thermo Fisher Scientific	Cat# A32723; RRID:AB_2633275
Alexa Fluor 594 Goat anti-Rabbit IgG (H+L)	Thermo Fisher Scientific	Cat# A32740; RRID:AB_2762824

**Biological samples**		

Human cortical brain tissue	Mesulam Center for Cognitive Neurology and Alzheimer’s Disease, Northwestern University	https://www.brain.northwestern.edu/

**Chemicals, peptides, and recombinant proteins**		

P110 and vehicle peptide TAT	Ontores Company	Cat# P104966

**Critical commercial assays**		

FD NeuroSilver Kit	FD NeuroTechnologies, Inc	Cat#PK301
Seahorse XF Cell Mito Stress Test kit	Agilent	Cat#103015
SynPer Synaptosome Isolation Reagent	ThermoFisher	Cat# 87793
MDA kit	Sigma Aldrich	Cat# MAK085
Duolink *In Situ* Red Starter Kit	Sigma Aldrich	Cat# DUO92101

**Experimental models**: Organisms/strains		

Traumatic Injury Overpressure Chamber	Louis Stokes Cleveland Veterans Association	N/A

**Software and algorithms**		

Any-maze behavior tracking software	Stoelting	https://www.any-maze.com/
GraphPad Prism	GraphPad Software, Inc.	https://www.graphpad.com/
ImageJ/Fiji	National Institute of Health, Bethesda, MD	https://imagej.nih.gov/ij
ZEN	ZEISS	https://www.zeiss.com/microscopy/en/products/software/zeiss-zen.html

### Experimental model and study participant details

#### Animals

7-week-old male and female C57BL/6J mice were obtained from The Jackson Laboratory and acclimated to the animal facility for 1 week prior to manipulation. Mice were group-housed with water and food provided *ad libitum* under controlled temperature, humidity, and light (12-h light/dark cycle) conditions. All animal procedures were performed in accordance with the protocol approved by the Louis Stokes Cleveland Veterans Affairs (VA) Medical Center Institutional Animal Care and Use Committee.

#### Human subjects

Tissue samples from human brain were provided by the Northwestern University Alzheimer’s Disease Research Center brain bank, through coordination with the Cleveland Alzheimer’s Disease Research Center Translational Therapeutics Core, and under approved study protocols. Written consent was provided by all donors for use of the brain tissues. Donor ages averaged 81 ± 5 (group 1), 83 ± 7 (group 2) and 85 ± 3 years old (group 3). The patient history of TBI, and additional age information is reported in the Supplemental Information (Table S1).

### Method details

#### *In vivo* multimodal traumatic brain injury

This procedure was conducted as previously described (*9,14–17,19-22*). 8-week-old mice were anesthetized with ketamine/xylazine (100/10 mg/kg) via intraperitoneal (i.p.) injection. Mice were placed in a padded, cylindrical shield protecting their body, leaving the head exposed and untethered. They were then secured in an enclosed chamber constructed from an air tank partitioned into two sides and separated by a port covered by a mylar membrane. The pressure in the side not containing the mouse was increased to cause membrane rupture at 20 pounds per square inch (PSI), which generates a ∼1–2 ms jet airflow of 137.9 ± 13.79 kPa that passes through the animal’s head. The jet of air produced upon membrane rupture provides a concussive injury, which is followed by acceleration/deceleration of the head and then exposure to the ensuing blast wave within an enclosed space.

#### P110 treatment

The P110 and control peptide TAT were synthesized by Ontores company (Hangzhou, China) (Product: P104966, Lot number: ON120414SF-01). The peptides were synthesized as one polypeptide with TAT_47–57_ carrier in the following order: N-terminus–TAT–spacer (Gly-Gly)–cargo (Drp149–55)–C-terminus. The C-termini of the peptides were modified to C (O)-NH2 using Rink Amide AM resin to increase stability. Peptides were analyzed by analytical reverse-phase high-pressure liquid chromatography (RP-HPLC) and matrix-assisted laser desorption/ionization (MALDI) mass spectrometry (MS) and purified by preparative RP-HPLC. The purity of peptides was >90% when measured by RP-HPLC Chromatogram. Lyophilized peptides were stored at −80°C and dissolved in sterile water before use.

In our previous studies, we found that P110 treatment in the dosage of 0.5–3 mg/kg/day range was effective in various disease models.^12^^,^^27^^,^^32^^,^^33^^,^^34^ We found that P110 at 1.5 mg/kg/day was protective in our TBI mouse model. Thus, in the current study, mice were treated with peptide P110 or control peptide TAT via i.p. injection at a dose of 1.5 mg/kg/day for 2 weeks after injury, beginning 3 h after injury. Treatment was then ceased, and mice were allowed to age. In the case of the “delayed treatment” cohort, treatment with either P110 peptide or vehicle was initiated at 8 months post-injury for 4 weeks at the same dose.

#### Western blotting

Tissues were flash frozen in liquid nitrogen after rapid dissection on ice. They were then homogenized in radioimmunoprecipitation assay (RIPA) buffer (Sigma-Aldrich R0278) containing protease and phosphatase inhibitors. Lysates were centrifuged for 30 min at 18,000xg at 4°C. Protein concentration of the supernatant was determined using the bicinchoninic acid (BCA) protein assay kit (Thermo Scientific A53225). Samples were prepared with 2x Laemmli sample buffer (Bio-Rad Laboratories 1610737) containing beta-mercaptoethanol and heated at 100°C for 5 min. Proteins were resolved in 4%–20% Criterion TGX Stain-Free gels (Bio-Rad Laboratories 5678095) and transferred onto 0.2 μm polyvinylidene fluoride (PVDF) membranes. Membranes were blocked using casein solution (Thermo Scientific 37528) for 1 h, and then incubated overnight at 4°C with the following primary antibodies, diluted in casein, to probe target proteins: Drp1 (BD Transduction 611113, 1:2000), phospho-Drp1 S616 (Cell Signaling Technology 3455S, 1:1000), Fis1 (Proteintech 10956-1-AP, 1:1000) and GAPDH (Cell Signaling Technology 2118s, 1:5000). Membranes were then rinsed 3 × 5 min in tris-buffered saline with tween 20 and incubated in horseradish-peroxidase-conjugated secondary antibodies diluted in casein solution for 1 h at room temperature. SuperSignalTM West Femto Maximum Sensitivity Substrate (Thermo Scientific 34096) was used to develop membranes, and Image Lab Software (Bio-Rad Laboratories, Inc) was used to analyze bands.

#### Behavioral testing

All behavioral testing was performed and scored in a randomized and blinded manner. All animals are accounted for. The novel object recognition (NOR) task was performed as previously described.^85^ Briefly, animals were habituated in the open field box for 5 min each. During this time, they were recorded and tracked using AnyMaze software to analyze parameters such as average speed and time spent in the center of the box, as a measure of anxiety-like behavior. 24 h later, animals were placed in the same box with two identical objects and allowed to freely explore the objects for a total of 20 s. 24 h later, one object was replaced with a new, different object and animals were again allowed to freely explore the objects for a total of 20 s “Discrimination index” was calculated as (time with novel object – time with familiar object)/(time with novel object + time with familiar object). The light/dark assay was performed, as previously described.^86^ Briefly, mice were placed in a box that was 50% exposed and 50% enclosed for 5 min and monitored using AnyMaze tracking software. Increased time spent exploring the open, “light” area was indicative of a lower anxiety-like phenotype. We also used the forced swim test and tail suspension test as measures of depressive-like behavior, as previously described.^87^^,^^88^ Briefly, mice were placed in a large tub of water and monitored for escape-oriented behaviors over 5 min. The time each mouse spent immobile was quantified, and longer immobility was interpreted as depressive-like behavior.

#### Transmission electron microscopy

Mice were euthanized by transcardial perfusion with 4% paraformaldehyde in phosphate-buffered saline (PBS) solution. Free-floating brain sections (40μM thick) were washed with PBS and fixed in quarter-strength Karnovsky’s fixative solution for 2 h at room temperature. After washing, specimens were postfixed for 2 h in an unbuffered 1:1 mixture of 2% osmium tetroxide and 3% potassium ferrocyanide. After rinsing with distilled water, specimens were soaked overnight in an acidified solution of 0.25% uranyl acetate. After another rinse in distilled water, specimens were dehydrated in ascending concentrations of ethanol, passed through propylene oxide, and embedded in EMbed 812 embedding media (Electron Microscopy Sciences, Hatfield, PA). Thin sections (70 nm) were cut on an RMC MT6000-XL ultramicrotome. These were mounted on Gilder square 300 mesh nickel grids (Electron Microscopy Sciences) and then sequentially stained with acidified methanolic uranyl acetate, followed by a modification of Sato’s triple lead stain. These were coated on a Denton DV-401 carbon coater (Denton Vacuum LLC) and examined in an FEI Tecnai Spirit (T12) with a Gatan US4000 4k × 4k resolution charge-coupled device (CCD).

#### Mitochondrial analysis

TEM images were collected from randomly selected neurons within the hippocampus. Mitochondria within each cell were traced on FIJI software, and particle analysis was completed on each image to determine Feret diameter and minimum diameter. The ratio between Feret and minimum diameter was then calculated to determine the aspect ratio of each mitochondrion.^38^ Aspect ratios from mitochondria of each mouse (approximately 700–1000 per mouse) were averaged, and subject to two-way ANOVA to determine the significance of the interaction between TBI and P110 treatment.

#### Synaptosome isolation

Animals were rapidly euthanized by cervical dislocation, and hippocampal and cortical tissues were dissected on ice. We determined that euthanasia by CO_2_ asphyxiation altered mitochondrial bioenergetics (Figure S8), and therefore rapid cervical dislocation was determined as the preferred method for isolation of live synaptosomes. Tissues were homogenized in Syn-Per reagent (ThermoFisher 87793) prepared with protease and phosphatase inhibitors using a glass Dounce homogenizer. Samples were centrifuged for 10 min at 1200xg at 4°C. Supernatant was then transferred to a fresh tube and centrifuged for 20 min at 15000xg at 4°C. Supernatant was discarded, and the pellet was resuspended in media of choice. For bioenergetic analysis, synaptosomes were resuspended in fortified XF RPMI, as described in the mitochondrial bioenergetics methods section below.

#### Mitochondrial bioenergetics

Synaptosomes were resuspended in XF RPMI (Agilent 103681), fortified with 1 mM pyruvate, 2 mM glutamine, and 10 mM glucose. Samples were then plated on a 96-well cell microplate provided in the XFe96 Flux Pak (Agilent 102601), with 20μg per well. Plates were spun for 20 min at 2000xg at 4°C, and subsequently subjected to the Cell Mito Stress Test (Agilent 103015) using the Seahorse XFe96 Analyzer. Three readings of oxygen consumption rate from each segment of the test were averaged, and spare respiratory capacity was calculated as the difference between maximal and basal respiration.

#### Immunohistochemistry

Mice were euthanized by transcardial perfusion with 4% paraformaldehyde in phosphate-buffered saline (PBS) solution. Free-floating brain sections (40μM thick) were processed and stained with an FD NeuroSilver Kit (FD NeuroTechnologies, Columbia, MD). For neurogenesis experiments, mice were injected intraperitoneally with a single bolus of 5-bromodeoxyuridine (BrdU) at a dose of 150 mg/kg. For the acute survival experiments, mice were injected 24 h after TBI, and euthanized 2 weeks post-injury by transcardial perfusion. For the chronic survival experiments, mice were injected with BrdU 3 weeks before euthanasia. Brains were processed and cut into 40μM thick, free-floating sections, which were then probed with anti-BrdU primary antibody (Sigma-Aldrich SAB4700630), followed by a biotin-conjugated secondary antibody (Vector Laboratories BA-2000). Sections were developed using diaminobenzidine and counterstained using hematoxylin to visualize anatomical structures. IgG extravasation staining was performed as described previously.^17^ BODIPY staining was performed to visualize lipid droplets as described previously.^68^ For 4-HNE staining, free-floating sections were subjected to antigen retrieval at 98°C in 10 mM sodium citrate buffer (pH 6.0). Thereafter, sections were blocked in 5% normal goat serum (S-1000, Vector Laboratories) and incubated overnight with rabbit anti-HNE (1:500, HNE11S, Alpha diagnostic International). On the following day, sections were incubated with goat anti-rabbit Alexa 594 (1:300; A32740, Invitrogen) for 2 h at room temperature, washed in PBS, and mounted using an antifade aqueous media (Vectashield Plus with DAPI, Vector Laboratories). Finally, the images were acquired on LSM 880, and mean fluorescence intensities were quantified with Fiji ImageJ software. Iba1 and GFAP immunofluorescent staining were performed using rabbit anti-Iba1 (Wako 019–19741) and mouse anti-GFAP (Thermo Fischer MA5-12023), followed with Alexa Fluor Plus 488 and 594 secondary antibodies.

#### TBARS assay of MDA

MDA was quantified with lipid peroxidation assay kit per the manufacturer’s instructions (MAK085, Sigma-Aldrich). Tissue was homogenized in lysis buffer containing butyrate hydroxytoluene, centrifuged, and supernatant was collected. Thiobarbituric acid was added to samples, incubated at 95°C for 60 min, and then cooled on ice for 10 min. Absorbance was read at 532 nm and concentrations were interpolated from a standard curve and normalized to the protein content of samples.

#### Co-immunoprecipitation of Drp1/Fis1 in TBI mouse model

Mouse cerebella was lysed in a total cell lysate buffer (50 mM Tris-HCl, pH 7.5, 150 mM NaCl, 1% Triton X-100, and protease inhibitor). Total lysates with 800 μg protein for each group were incubated with the anti-Drp1 antibody (BD Biosciences, 611738) overnight at 4°C followed by the addition of protein A/G beads (Santa Cruz, sc-2003) for 2 h at 4°C. Immunoprecipitates were washed four times with cell lysate buffer and were analyzed by western blot using anti-Drp1 (1:1000) and anti-Fis1 (Proteintech, 10956-1-AP. 1:1000) antibodies.

#### *In situ* proximity ligation assay (PLA)

PLA was performed using the Duolink *In Situ* Red Starter Kit (mouse/rabbit, DUO92101, Sigma). Briefly, brain sections were permeabilized and blocked with PLA blocking buffer for 1 h at 37°C and incubated with the anti-Drp1 (1:1000) and anti-Fis1 (1:1000) antibody overnight at 4°C. The sections were then incubated with the PLA probes (Anti-Rabbit PLUS and Anti-Mouse MINUS) for 1 h at 37°C, followed by the ligation and amplification steps. The PLA signal was visible as a distinct fluorescent spot and analyzed by confocal microscopy (Fluoview FV3000, Olympus). The number and size of the fluorescent signals was quantitated using NIH ImageJ software.

### Quantification and statistical analysis

All statistical analyses were performed using GraphPad Prism, version 9.0.0 (GraphPad Software, Inc.), and all quantifications and analyses were performed in a blinded manner. For animal experiments comparing injury groups, the groups were compared using independent samples t tests. For animal experiments with both injury and treatment experimental groups, two-way ANOVA models were estimated in which the interaction of injury (TBI vs. Sham) and treatment (P110 vs. Vehicle) was the effect of interest, assessing whether the effect of TBI was significantly altered by P110 treatment, and results were reported as an “interaction *p*-value.” When a significant interaction effect was detected, Tukey’s post hoc tests were performed and significant pairwise differences between injury and treatment groups identified. Human subject results were analyzed using one-way ANOVA with Tukey post hoc pairwise comparisons. Data are presented as mean ± SEM, and individual data points represent individual samples or animals. Details of statistical analysis can be found in the figure legends. Significance was determined at *p* value below 0.05.

## Supplemental information

Document S1. Figures S1–S8 and Table S1

Document S2. Article plus supplemental information
