# Supplementary material for: Acutely blocking excessive mitochondrial fission prevents chronic neurodegeneration after traumatic brain injury
Source: Cell Rep Med. 2024 Sep 5;5(9):101715. doi: 10.1016/j.xcrm.2024.101715 (PMC11525032; doi:10.1016/j.xcrm.2024.101715)
Supplement: Document S1. Figures S1–S8 and Table S1 [file mmc1.pdf]

Cell Reports Medicine, Volume 5

Supplemental information

Acutely blocking excessive mitochondrial fission prevents chronic neurodegeneration after traumatic brain injury

Preethy S. Sridharan, Yeojung Koh, Emiko Miller, Di Hu, Suwarna Chakraborty, Sunil Jamuna Tripathi, Teresa R. Kee, Kalyani Chaubey, Edwin Vázquez-Rosa, Sarah Barker, Hui Liu, Rose A. León-Alvarado, Kathryn Franke, Coral J. Cintrón-Pérez, Matasha Dhar, Min-Kyoo Shin, Margaret E. Flanagan, Rudolph J. Castellani, Tamar Gefen, Marina Bykova, Lijun Dou, Feixiong Cheng, Brigid M. Wilson, Hisashi Fujioka, David E. Kang, Jung-A.A. Woo, Bindu D. Paul, Xin Qi, and Andrew A. Pieper

Supplemental Information

Acutely blocking excessive mitochondrial fission prevents chronic neurodegeneration after traumatic brain injury

Preethy S. Sridharan, Yeojung Koh, Emiko Miller, Di Hu, Suwarna Chakraborty, Sunil Jamuna Tripathi, Teresa R. Kee, Kalyani Chaubey, Edwin Vázquez-Rosa, Sarah Barker, Hui Liu, Rose A. León-Alvarado, Kathryn Franke, Coral J. Cintrón-Pérez, Matasha Dhar, Min-Kyoo Shin, Margaret E. Flanagan, Rudolph J. Castellani, Tamar Gefen, Marina Bykova, Lijun Dou, Feixiong Cheng, Brigid M. Wilson, Hisashi Fujioka, David E. Kang, Jung-A A. Woo, Bindu D. Paul, Xin Qi, Andrew A. Pieper

Title: Acutely blocking excessive mitochondrial fission prevents chronic neurodegeneration after traumatic brain injury

Author List: Preethy S. Sridharan[1,2,3,4,5], Yeojung Koh[1,2,3,4,6], Emiko Miller[1,2,3,4,5], Di Hu[7], Suwarna Chakraborty[8], Sunil Jamuna Tripathi[8], Teresa R. Kee[6,9], Kalyani Chaubey[1,2,3,4], Edwin Vázquez-Rosa[1,2,3,4], Sarah Barker[1,2,3,4,6], Hui Liu[1,2,3,4], Rose A. León-Alvarado[1,2,3,4,10], Kathryn Franke[1,2,3,4], Coral J. Cintrón-Pérez[1,2,3,4], Matasha Dhar[1,2,3,4], Min-Kyoo Shin[1,2,3,4,11], Margaret E. Flanagan[12,13,14], Rudolph J. Castellani[15,16], Tamar Gefen[15,17], Marina Bykova[18,19], Lijun Dou[19], Feixiong Cheng[19], Brigid M. Wilson[3,20], Hisashi Fujioka[21], David E. Kang[6,9,20], Jung-A A. Woo[6,9], Bindu D. Paul[8,22,23,24], Xin Qi[7,*], Andrew A. Pieper[1,2,3,4,5,6,25,*]

This document contains 8 supplementary figures and 1 supplemental table.

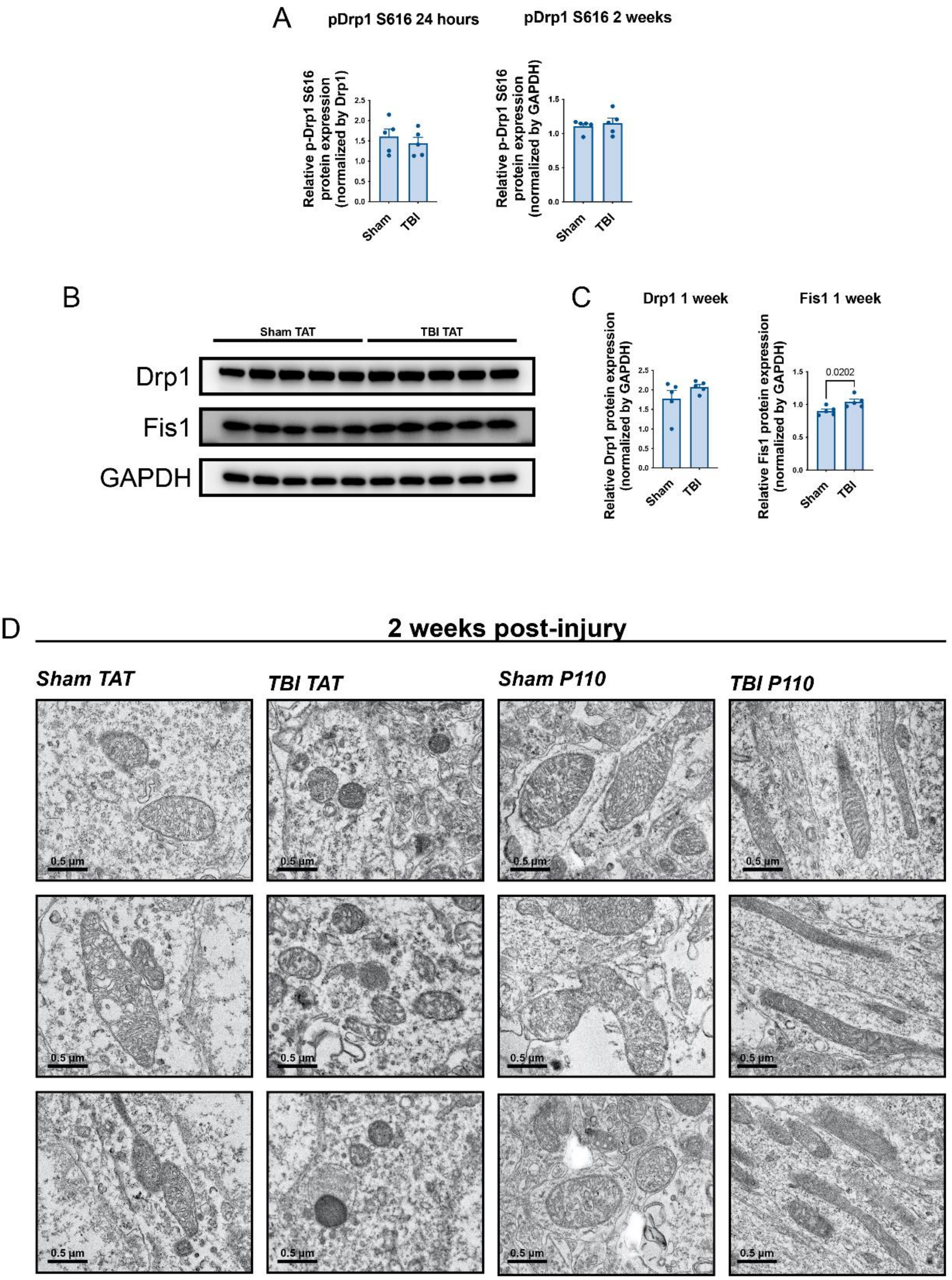

Figure S1. Stable expression and phosphorylation of Drp1, elevated Fis1, and increased mitochondrial fragmentation after TBI. Related to Figure 1. (A) pDrp1 (serine 616) normalized to Drp1 expression 24 hours and 2 weeks after TBI (n=5 mice/group, two-tailed student's t-test). (B) Drp1 protein expression is not affected by TBI at 1 week post injury. Fis1 levels are significantly elevated (n=5 mice/group, two-tailed student's t-test**).** (C) Additional representative images of hippocampal mitochondria 2 weeks post-injury.

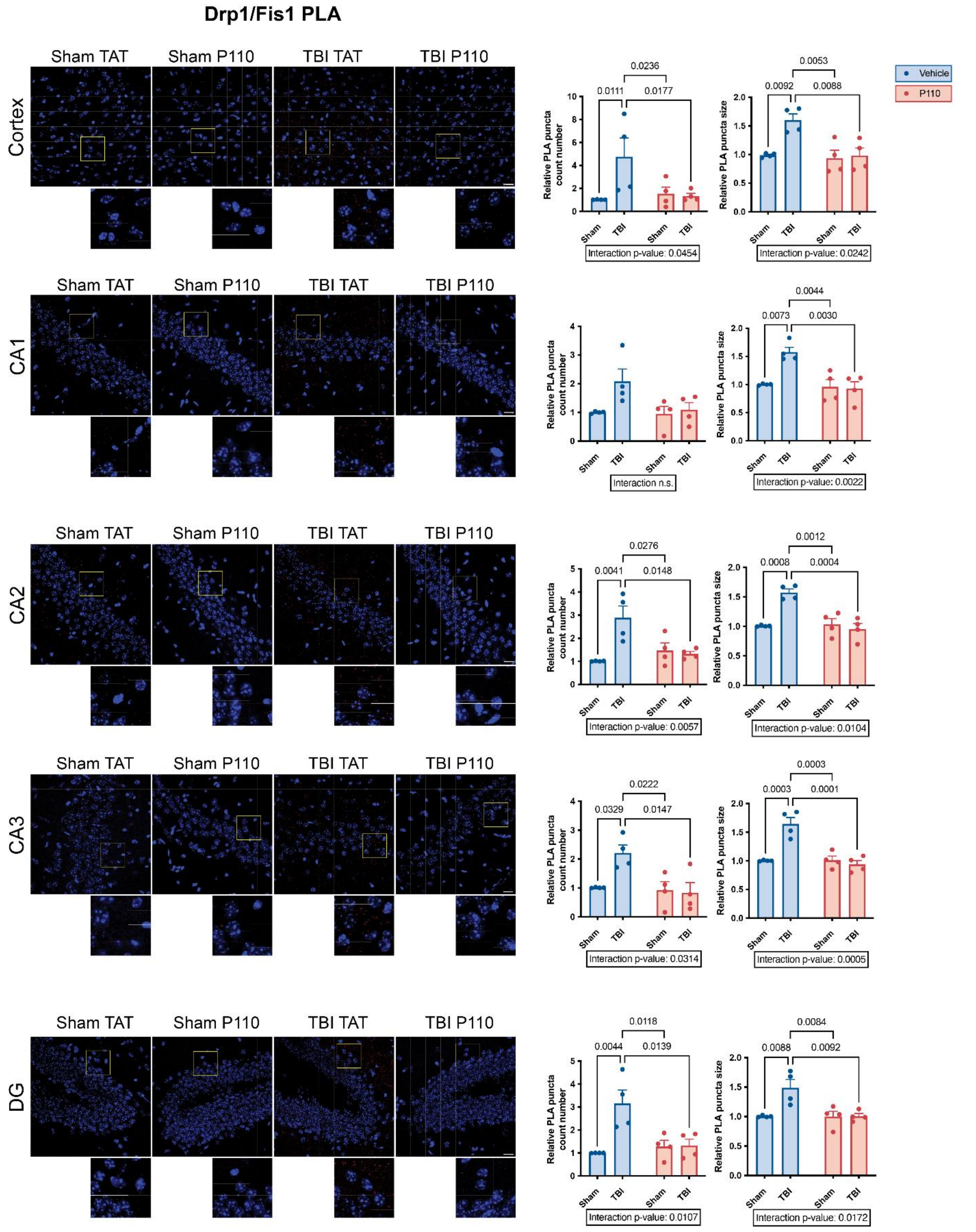

Figure S2. Increased Drp1-Fis1 interaction in hippocampal tissues after traumatic brain injury is attenuated with P110 treatment. Related to Figure 1. Representative images and quantification of proximity ligation assay from cortex, and CA1, CA2, CA3, and dentate gyrus regions of hippocampus. Respective quantification of puncta count number and puncta size in each region (n=4 mice/group. Two-way ANOVA and Tukey's post hoc analysis).

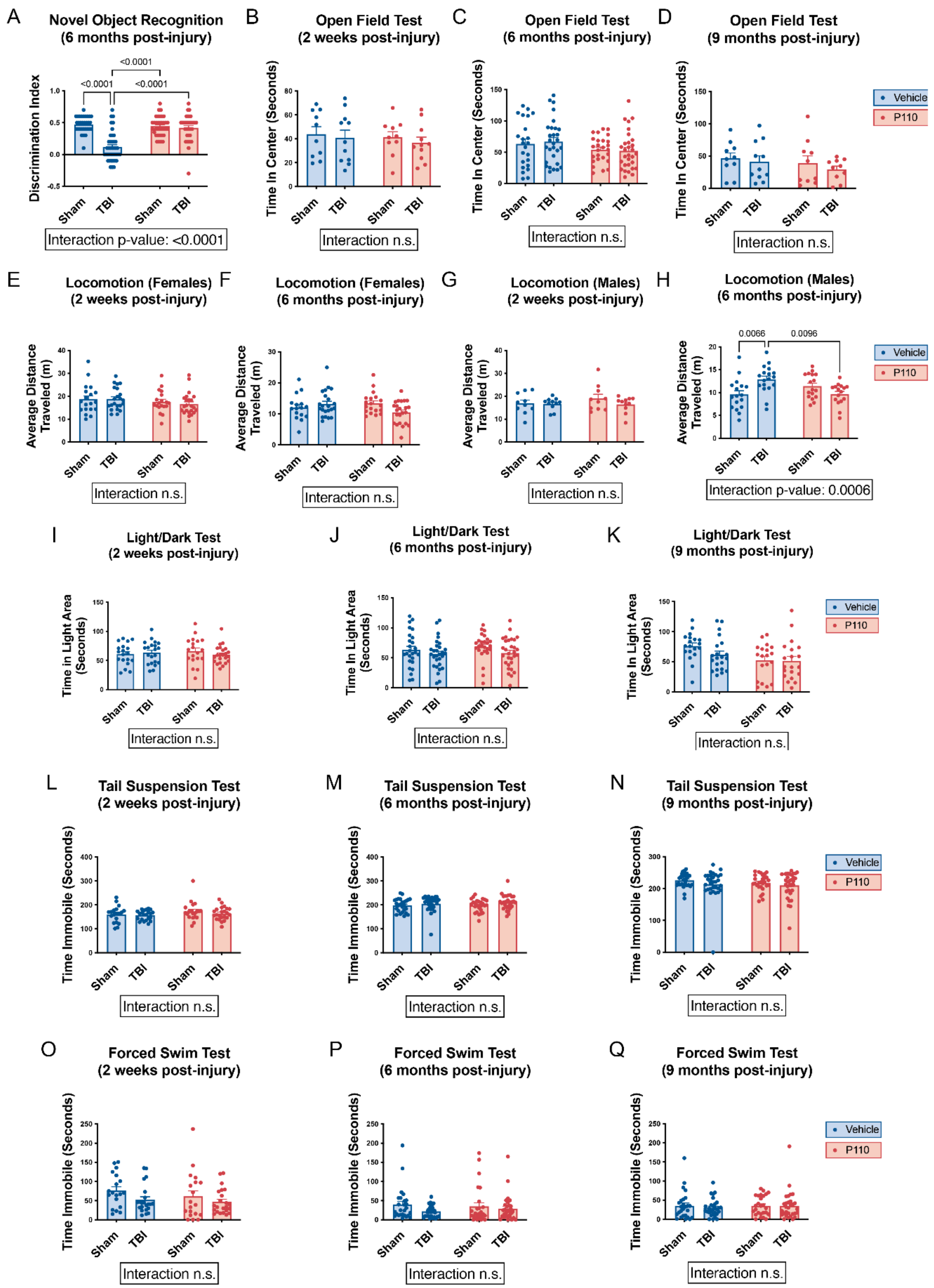

Figure S3. Absence of anxiety-like or depressive-like behavior after TBI. Related to Figure 1. (A) Novel object recognition task at 6 months post-injury shows significant cognitive impairment, which is prevented by early transient P110 treatment (n=35-40 mice/group. Two-way ANOVA and Tukey's post hoc analysis)**.** (B to D) Open

field test reveals no acute or chronic changes in anxiety-like behavior after brain injury (n=35-40 mice/group. Two-way ANOVA and Tukey's post hoc analysis). (E to H) Locomotor activity, measured as total distance traveled in the open field test, reveals a male-specific hyperlocomotion 6 months post brain injury. This effect is prevented by early transient P110 treatment (n=10-20 mice/group. Two-way ANOVA and Tukey's post hoc analysis). (I to K) Light/dark test reveals no acute or chronic changes in anxiety-like behavior after brain injury (n=35-40 mice/group. Two-way ANOVA and Tukey's post hoc analysis). (L to N) Tail suspension test reveals no acute or chronic changes in depressive-like behavior after brain injury (n=35-40 mice/group. Two-way ANOVA and Tukey's post hoc analysis). (O to Q) Forced swim test reveals no acute or chronic changes in depressive-like behavior after brain injury (n=35-40 mice/group. Two-way ANOVA and Tukey's post hoc analysis). Data are the mean ± SEM. Two-way ANOVA tests were used for interaction between injury effect and treatment effect.

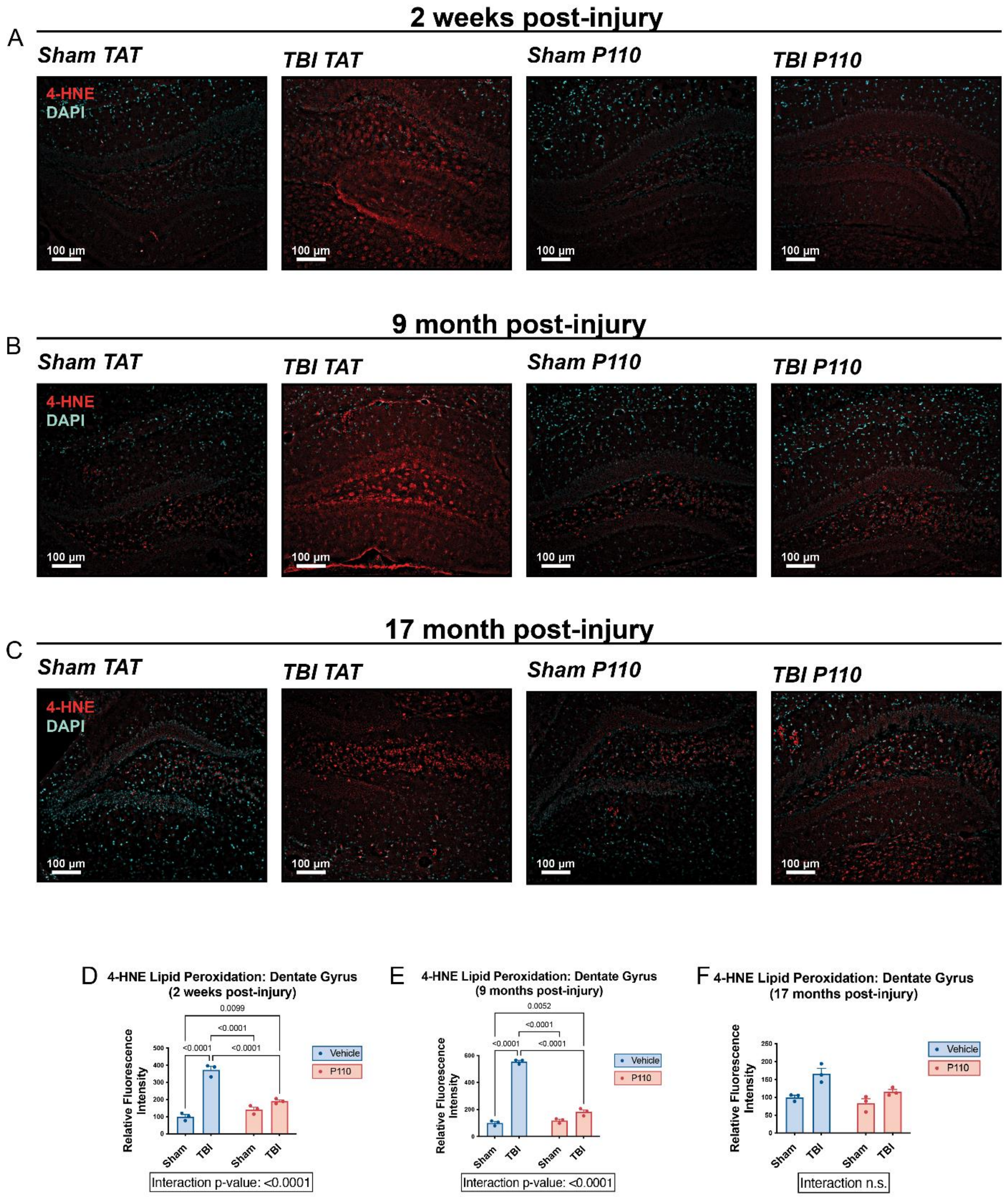

Figure S4. Prevention of increased hippocampal lipid peroxidation and lipid droplet accumulation in the dentate gyrus of the hippocampus after TBI by acute inhibition of pathologically excessive mitochondrial fission. Related to Figure 3. (A to C) Representative 4-HNE staining of the hippocampal dentate gyrus region shows increased lipid peroxidation at 2 weeks, 9 months, and 17 months post-injury. Early transient P110 treatment strategy prevents 4-HNE elevation. (D to F) Quantification of lipid peroxidation from dentate gyrus region of hippocampus (n=3 mice/group. Two-way ANOVA and Tukey's post hoc analysis).

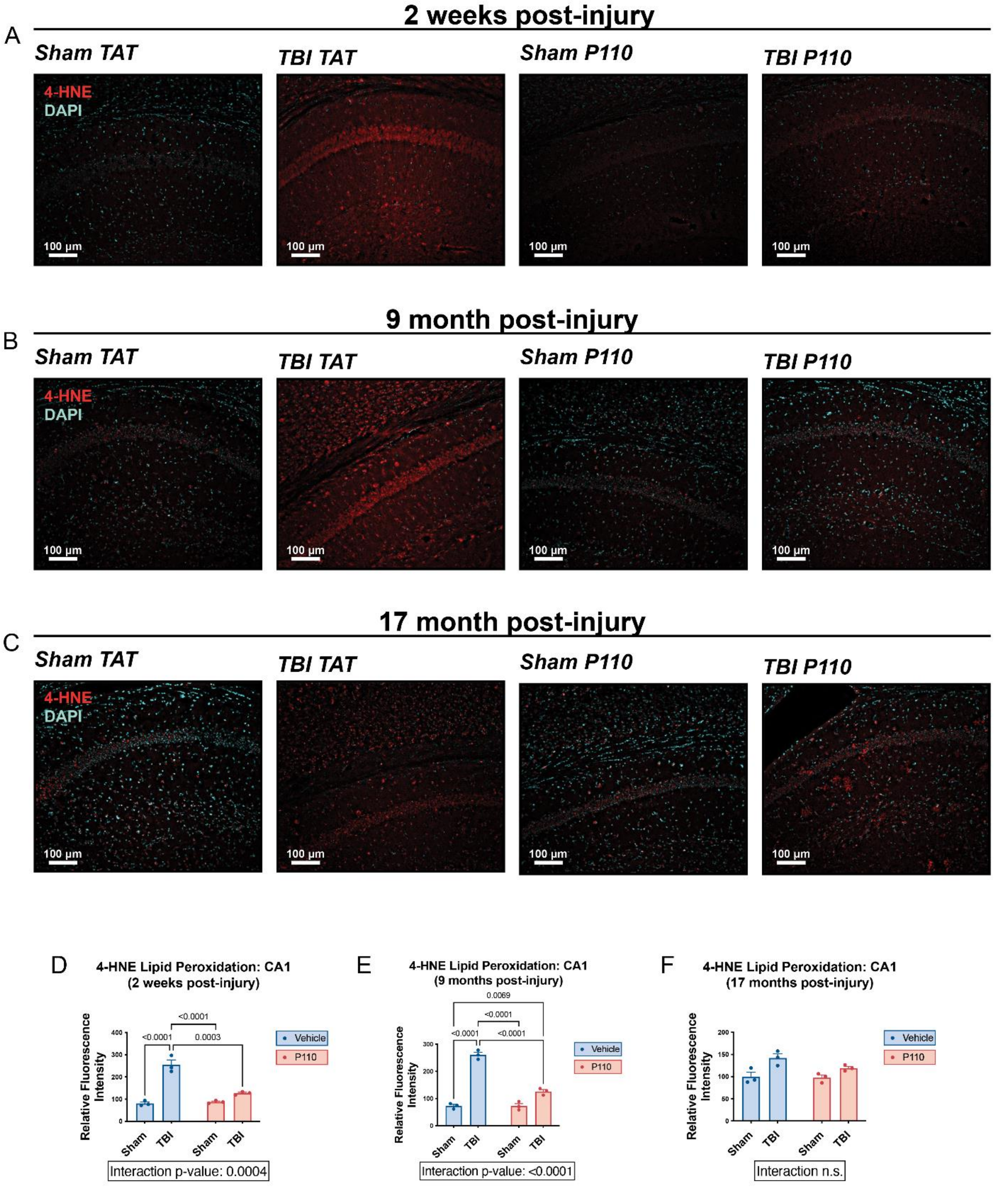

Figure S5. Prevention of increased hippocampal lipid peroxidation and lipid droplet accumulation in the CA1 region of the hippocampus after TBI by acute inhibition of pathologically excessive mitochondrial fission. Related to Figure 3. (A to C) Representative 4-HNE staining of the hippocampal CA1 region shows increased lipid peroxidation at 2 weeks, 9 months, and 17 months post-injury. Early transient P110 treatment strategy prevents 4-HNE elevation. (D to F) Quantification of lipid peroxidation from CA1 region of hippocampus (n=3 mice/group. Two-way ANOVA and Tukey's post hoc analysis).

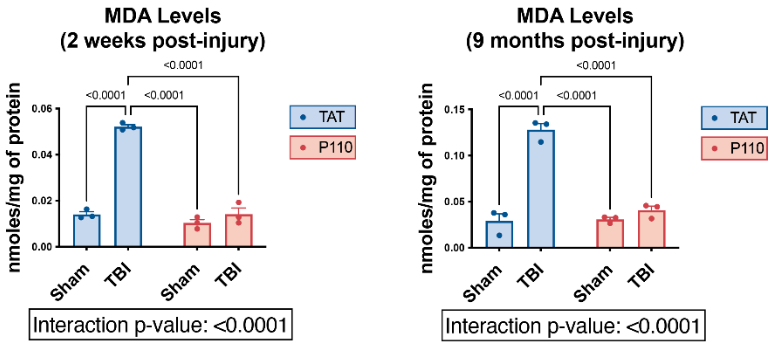

Figure S6. Prevention of chronic MDA elevation after TBI by acute inhibition of pathologically excessive mitochondrial fission. Related to Figure 3. Quantification of MDA levels obtained from flash frozen brain tissue at 2 weeks and 9 months post-injury (n=3 mice/group. Two-way ANOVA and Tukey's post hoc analysis).

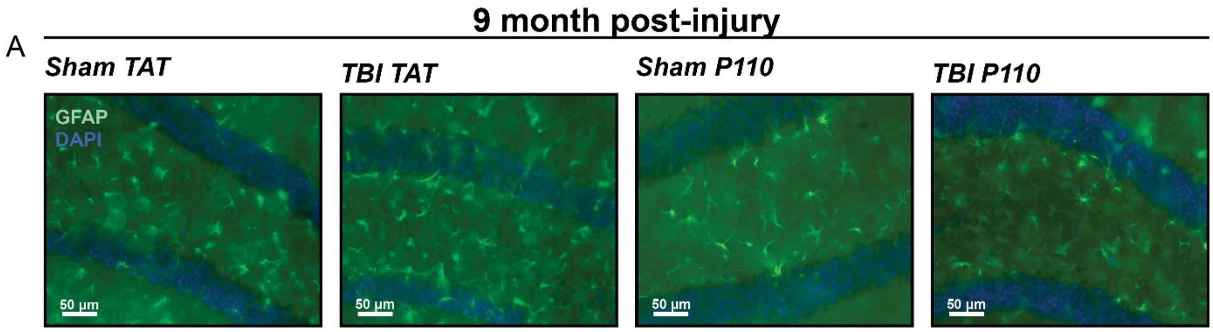

Figure S7. Absence of chronic astrocytic activation after TBI. Related to Figure 7. (A) Representative images of GFAP+ cells in the hippocampus of mice 9 months post-injury.

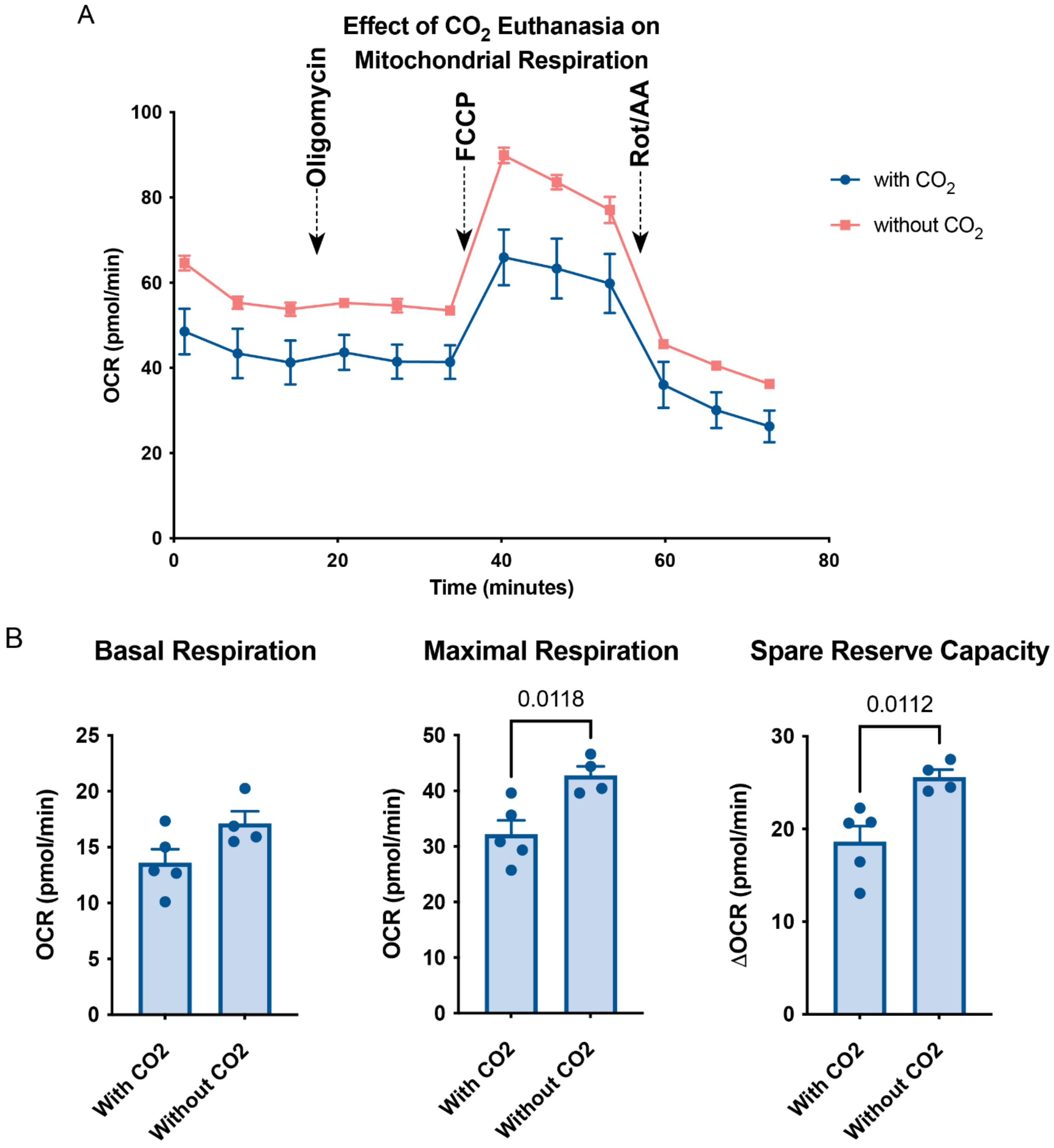

Figure S8. Impaired mitochondrial bioenergetics in isolates synaptosomes by CO_2 asphyxiation euthanasia. Related to Figure 2, as described in Methods. (A) Oxygen consumption curve shows reduced mitochondrial respiration after CO_2 euthanasia. (B) Quantification of oxygen consumption shows no statistically significant change in basal respiration, but significant reductions in maximal respiration and spare respiratory capacity (n=5 samples/group, two-tailed student's t-test).

Clinical Group	Post-Mortem Interval (Hours)	Age at Death (Years)	Sex	Race/Ethnicity	Neuropathologic Diagnosis
Group 1: No Cognitive Impairment (NCI)					
Subject 1	16	87	F	Caucasian	ADNC LOW
Subject 2	5	80	M	Caucasian	ADNC LOW
Subject 3	13	76	F	Caucasian	ADNC LOW
Group 2: Dementia of Alzheimer's Type without history of TBI (DAT)					
Subject 1	71	89	F	Caucasian	ADNC HIGH
Subject 2	7	82	F	Caucasian	ADNC HIGH
Subject 3	5	85	F	Caucasian	ADNC HIGH
Group 3: Dementia of Alzheimer's Type with history of TBI (DAT + TBI)					
Subject 1	19	75	F	Caucasian	ADNC HIGH
Subject 2	9	86	M	Caucasian	ADNC HIGH
Subject 3	24	89	F	Caucasian	ADNC HIGH

Supplementary Table 1. Patient demographic information for human postmortem tissue used in Fig. 1B.
